# Hospital and Institutional News

**Published:** 1917-11-24

**Authors:** 


### HOSPITAL AND INSTITUTIONAL NEWS.

### THE BRITISH HOSPITALS ASSOCIATION.

In response to an invitation from Sir Charles Harris, Financial Secretary of the War Office, a deputation from the Council of the British Hospitals Association, consisting of the chairman (Mr. H. Wade Deacon), the Rev. G. B. Cronshaw, Mr. E. W. Morris, Mr. G. Q. Roberts, and the honorary secretaries, was received at the War Office on Friday, November 16, to discuss the questions arising out of the resolution passed at the conference held at Westminster Hospital (see The Hospital, October 27, pages 79 *et seq.*) in reference to the treatment of wounded sailors and soldiers. The Financial Secretary extended a very sympathetic reception to the deputation, and expressed keen appreciation of the wonderful work the voluntary hospitals have done for the soldiers. In the result, at Sir Charles Harris' suggestion, the Council of the British Hospitals Association was requested to appoint two representatives to meet representatives of the War Office to discuss the question further. This course was adopted by the Council at their meeting on Friday afternoon at St. Bartholomew's Hospital, when the Rev. G. B. Cronshaw and Mr. E. W. Morris were appointed representatives to confer with the War Office authorities. In view of the conferences now being held in London between the hospitals, the Council has decided to defer arranging for a deputation to the Ministry of Pensions in reference to the subject of the treatment of discharged soldiers, to which the second resolution passed at the Westminster Hospital related, until after the results of the conferences are known.

### THE ORTHOPÆDIC ANNEXE.

When Sir Arthur S. T. Griffith Boscawen, M.P., Parliamentary Secretary to the Ministry of Pensions, opened last week the Training Centre in Charlotte Street, annexed to the Aberdeen Military Orthopædic Department, for the treatment of discharged disabled men, a new institutional department became established in Scotland. The annexe consists of rooms for the electrical and massage treatment of disabled sailors and soldiers who have returned to civil life. The hours and staff are supposed to be ready to suit the patients' convenience, so that the treatment received in hospital may not be interrupted by their professional occupation. In this way the ideal of an out-patient department as a consultation centre is realised for one class at least.

### THE RUSSIAN RED CROSS.

An interesting account of the work of the Russian Red Cross was given at a recent lecture at King's College, London, by Mr. Mouravieff Apostol, under the auspices of the United Russia Societies' Association. The chair was taken by Sir Arthur Stanley, who spoke of the services rendered by Mr. Mouravieff Apostol in the establishment of the excellent Russian Hospital in South Audley Street. Mr. Apostol pointed out that the fact that the Russian Red Cross was quite unprepared at the commencement of the war was a proof that Russia had made no real preparation for fighting. In the original mobilisation plans the Russian Red Cross was to provide ten advanced units or flying columns; there were now 118 of these units, and in addition there were 98 ambulance transport services. There were 85 movable hospitals in place of 33 originally, and the number of base hospitals had also been greatly increased. The Red Cross had further to equip and maintain the ambulance trains, and of these there were now 54, in addition to temporary "flying trains." The total number of Red Cross nurses on active service and in the reserve at the beginning was 6,000, but by last January the number had increased to 26,000. The difficulties of transporting the wounded and also the refugees from the invaded districts had been enormous, but for the most part they had been successfully overcome.

### IN PLACE OF THE PEACE-TIME DINNER.

The annual festival dinner, because of the war, has been promoted in some instances to a luncheon, and, since even a luncheon has seemed to some an excessive meal to take officially in these times, the hospital dinner has turned into a tea. Whether this means a high-tea or not it would surely be irrelevant to inquire. The humour of the situation cannot detract from its seriousness. We, therefore, urge all people of public spirit who have been invited to spend an hour at the Drapers' Hall, Throgmorton Street, on December 6, at 4.45 p.m., to go. They will learn from Lord Knutsford and others what air-raids mean to a hospital for accidents in East London. The elusive Gothas and the cumbrous Zeppelins find the Poplar Hospital in their path to Piccadilly. And since their path is a war-path indifferent to its victims, the Poplar Hospital is apt to have a busy and an anxious time. Workmen in industrial centres have mostly agreed to make their weekly penny gift to their local hospital twopence. If professional and business people in London on whom the Poplar Hospital largely relies will do the same, it can continue its work. Can Londoners do this? It should be added that the tea generously provided by the Drapers' Company will take place at four o'clock. The advantage of this plan is that anyone who may be late for the tea will be in time for the meeting.

### THE GROWTH OF LOCAL LABORATORIES.

Sir William Osler, who once gave a spirited address upon the new departments which provincial hospitals should develop as opportunity was found, will have noted the new fund established by Mr. R. B. Loder for the creation of a bacteriological laboratory at Northampton General Hospital. Up to now Birmingham and London have received all specimens requiring expert analysis, in consequence of which the specimens in transit have run serious risks, and time is lost, in both cases, to the disadvantage of everyone in Northampton. Mr. Loder has set the financial scheme in motion by collecting gifts from others and adding donations of his own. Without the laboratory, diagnosis must often be based on evidence obtained elsewhere, and there is no local means to prepare vaccines. We hope that the laboratory fund will quickly be raised, and that the example of Northampton will be followed by hospitals in other provincial centres of equal standing.

### GENEROUS GIFTS.

Two generous gifts were announced in the North of England last week. The first is the presentation of Sutton Hall, situated in the village of Sutton-under-Whitestonecliffe, four miles from Thirsk, in Yorkshire, by Mr. James H. Edwards, a Justice of the Peace for the North Riding, to the Sailors' and Firemen's Union, as a home of rest for worn-out seafarers. The hall will accommodate between 300 and 400 inmates. The second is the gift of the Westfield Estate, Lancaster, by members of the Storey family (who are industrial magnates in the town), as a nucleus of a series of villages for disabled sailors and soldiers. It is proposed to construct the village—which will be the first of its kind in the country—on the lines desired by Mr. T. H. Mawson, the well-known architect, who has gone to Salonika to prepare plans for the reconstruction of that city. Mr. Mawson has described his ideas for villages for the disabled in his book "An Imperial Obligation." The estate, upon which accommodation will be provided for 300 disabled men, half of whom will be the heads of families, will be laid out with workshops, houses, hostels for the single men, with communal kitchens, a church, and recreation grounds, gardens, and a public park with a bandstand. The existing mansion will be used as a club-house and social centre. It is believed that friends of fallen sailors and soldiers will bear the cost of erecting, as memorials, single houses or groups of houses in the village.

### AN INTERESTING BEQUEST.

HOSPITAL secretaries must always feel considerable curiosity as to the exact circumstances under which legacies are bequeathed to their hospitals. In many cases it is not very difficult to trace the reasons underlying the benefaction, as the testator is often a governor of the hospital. It sometimes happens, however, that no connection of any sort can be traced. A striking example of this is furnished by the legacy of £2,500 bequeathed to the Grosvenor Hospital for Women, Vincent Square, by Mr. George Samuel Weir. Neither Mr. Davidson, the secretary, nor any member of the board of management, had any knowledge of him, and the hospital's first acquaintance with his name occurred in the letter from a firm of solicitors at Liverpool, notifying the legacy. There was only one other hospital bequest in the will, and the reasons which led Mr. Weir to pick out the Grosvenor Hospital for a comparatively large gift must form an interesting speculation. It is often some slight circumstance, some knowledge of the good work done at the hospital, which has determined the legacy.

### A LOSS TO HAMPSTEAD HOSPITAL.

THE estate of Major Valentine Fleming, M.P., which has just been proved at £255,000, recalls the loss sustained by Hampstead General Hospital in the death of this gallant officer. Major Fleming, who lived at Pitt House, Hampstead, was elected chairman of the Hampstead Hospital shortly before the outbreak of the war; but as a Territorial officer he was immediately mobilised, and was never able to take up the duties of the chairmanship. Nevertheless, the Council of the hospital kept the post open, the deputy-chairman, Mr. C. W. Sawbridge, filling the gap, although he himself was well occupied with his duties as chairman of the Great Northern Central Hospital. The arrangement was in vain, and the sacrifice made by Major Fleming on the field of action means to the Hampstead Hospital the loss of a good friend.

### AN INQUIRY DEMANDED AT LAMBETH INFIRMARY.

LAMBETH BOARD OF GUARDIANS have decided to hold an inquiry "into the terms and conditions of employment, the emoluments, and the qualifications of the medical staff at the infirmary, and the administration at this institution, both lay and medical, from A to Z." It has been evident for some time that some dissatisfaction has existed, but who or what is to blame has not transpired. A committee has been considering the question of the lay administration at the infirmary, and in their latest report state that before submitting any suggestions on the subject, the board in committee should consider matters which had come under their observation, with a view to an expression of opinion as to the direction of the administration in future. The chairman, Mr. Frank Briant, L.C.C., whilst agreeing that the board should hold the inquiry, said that the time had come when pin-pricks aimed at officers should cease. The resolution involved more than administration: it reflected upon the care of the sick and upon the medical treatment at the infirmary, and he had never until that day heard of any founded case of failure to mete out proper treatment, whilst he did not feel inclined to accept rumour. The inquiry will have the effect, it is hoped, of clearing these rumours, and bringing about a more sympathetic and closer union between the lay and medical sides of the infirmary.

### CHRISTMAS ENTERTAINMENTS.

A LETTER written by the Food Controller's department in respect of Christmas festivities states that no order has been issued to prevent parties and entertainments being held. The view taken, however, is that all entertainments which lead to an undue consumption of food, and particularly of breadstuffs, should be discouraged. Such entertainments fall naturally into two classes—those in which amusement is the main consideration, and refreshments are merely incidental, and those in which a good meal for the poor is the main object. In the first instance, Lord Rhondda considers, refreshments should as far as possible be dispensed with; in the second, the food provided should consist wherever possible of non-essential foodstuffs, and care should be taken to avoid those articles of which there is a temporary shortage, such as tea. We are glad that the Food Controller advocates discretion in those matters. In the knowledge of most hospital workers there is a great deal of quite unnecessary junketing in hospitals, for instance, at Christmas-time. The cost of this is, of course, borne by money given specifically for the purpose; but the effect is distinctly bad and creates a quite undesirable feeling amongst the patients and staff that superfluity in catering is justifiable and possible. One good entertainment on Lord Rhondda's lines will appear to sensible people quite sufficient for each ward.

### FIRST-AID IN FACTORIES.

THE Home Secretary has made an Order, coming into force on December 1, requiring the provision of first-aid and ambulance arrangements in blast-furnaces, copper-mills, iron-mills, foundries, and metal-works. Its object is to provide speedy treatment for injuries sustained in the course of work, and thus to minimise the risks of sepsis, and prevent in the case of serious lesions the shock of a possibly long journey to the nearest hospital before relief can be obtained. The general scheme includes first-aid posts conveniently situated in different parts of the works, and, where more than 500 hands are employed, a central ambulance room. Each aid post must be in charge of some one person who is to be held responsible for the dressings, etc., being in good order, and whose name must be posted in a prominent position in the department concerned. Anyone with hospital experience must have realised the large amount of time lost owing to minor injuries that have become septic from neglect in the first instance, and at a time like the present, when labour of all kinds is at a premium and it is necessary that every man should be working with the highest possible efficiency, the benefits of such a provision cannot be overestimated.

### SPOTTED FEVER.

SUMMARISING in a memorandum issued last week the results of investigations made by the Local Government Board into the subject of cerebro-spinal (spotted) fever, Sir Arthur Newsholme, principal medical officer, says the disease may be contracted by kissing or by several persons eating or drinking out of the same vessel. "Inasmuch as during the prevalence of cerebro-spinal (spotted) fever a considerable proportion of the general public harbour meningococci, it is desirable that certain precautions should become customary," says the memorandum. "These precautions should include the careful use of handkerchiefs in sneezing and coughing and the occasional use of antiseptic gargles and sprays."

### "OUR DAY" IN INDIA.

THE preparations for a great celebration in aid of the Red Cross in India are being organised on a scale of true Oriental lavishness. The Viceroy has declared December 12 a public holiday, upon which special displays will be given by the Army, and races held by the Calcutta and Bombay Turf Clubs, the proceeds of which will be given to the funds. Maharajas are giving lakhs, and fractions of lakhs, of rupees, with prodigal generosity. Wealthy merchants are defraying the cost of millions of Union Jack button-hole flags—even India, it seems, has its flag days—sent out specially from England. Queen Mary has contributed a number of gifts, to be hidden in the recesses of "lucky bags," and no doubt fabulous sums will be paid for the privilege of diving therefor. Provincial Governors, Lieutenant-Governors, Commissioners, and all the hierarchy of Indian officialdom is entering whole-heartedly into the scheme. In view of the oft-repeated rumours of native discontent and unrest, it is reassuring to learn that the native States are making every effort to give the enterprise the success it so justly deserves.

### UNFOUNDED CHARGES AGAINST A HOSPITAL.

AT an inquest held at Battersea serious complaints were made against the medical treatment meted out to a patient at the West London Hospital. A man cut his left wrist whilst at work, and it was contended that when he was taken to the hospital, the doctor, instead of tying up the severed arteries, bandaged the wound, and told the man to go to his panel doctor. Some hours later the bleeding began again. Dr. Denis Lucey, senior house surgeon at the West London Hospital, said deceased had a punctured wound on the left wrist, which had stopped bleeding when he saw it. He had evidently cut a large artery, and he decided to try to find the end and tie it up, but that could not be done without an anæsthetic. On examination it was found that the man had heart disease, and therefore it was inadvisable to administer an anæsthetic. The wound was bound up, but later he returned as the bleeding had recommenced. A small subcutaneous injection of cocaine was given, and with some difficulty the bleeding points were found and tied. There were several severed arteries. As a few days later the wound became septic, the man was admitted to the hospital, eventually being discharged and treated as an out-patient. He was then admitted to St. James' Infirmary, Wandsworth, where he was under the care of Dr. Macormac, the medical superintendent; he was then suffering from erysipelas, death being due to fatty degeneration of the heart, following erysipelas. He thought the treatment given at the West London Hospital was perfectly correct. The jury, in returning a verdict of "Accidental death," expressed the opinion that no blame could be attached to the hospital.

### THE LATEST SCRAP OF PAPER.

WE quote, by itself, the following note and editorial comment from the October number of the *Searchlight*, the gazette of the 2nd Western General Hospital, Manchester, on the subject of "Raiding the R.A.M.C.," which has been lately discussed in the London newspapers. The editor of the *Searchlight* writes:—

"One of our correspondents puts the case very concisely in a letter to the member of Parliament for his constituency, a copy of which he has forwarded to us. He says: 'In the House of Commons on July 11, 1917, Mr. Macpherson, Under-Secretary for War, in reply to a question put by Mr. Harvey, M.P., said: Arrangements have been made by which any men who enlisted *with the express provision* that they should only be employed on R.A.M.C. work, would remain with the R.A.M.C.' I am one of those who joined the R.A.M.C. in 1915 before conscription was in vogue, and hold a certificate from the O.C. of my unit exempting me from all provisions of the Military Service Act, 1916. Last Friday I was one amongst one thousand R.A.M.C. men despatched from Blackpool to Warminster for transfer to the infantry. This action of the authorities is directly opposed to the statement above quoted, unless the phrase italicised is intended to enable the authorities to treat my attestation form as 'a scrap of paper.' . . . My enlistment was a voluntary act, and Mr. Macpherson's statement in the House should be a sufficient guarantee for my protection."

The editor of the *Searchlight* remarks: ". . . Our correspondent has since been sent to Salisbury Plain for training. The logic of [his] case is perfectly sound, and we await with interest the result of his appeal."

### THE "SPRINGBOK MAGAZINE."

Colonial war hospital gazettes are but little known outside their own circle in this country, and we have pleasure in introducing the *Springbok Magazine* to a wider range of readers. It is the publication of the South African Military Hospital, Richmond Park, Surrey, and claims attention from the uncles and the cousins and the aunts, to say nothing of the parents, of "our boys," presumably the South African wounded in England and in the field. Fiction is forbidden, and the editors undertake to "endeavour with all our strength to escape the charge of profanity." The November number contains "An Incident with the Field Ambulance," a "Pilgrimage to the Butte of Warlencourt," a list of the patients, a "list of the named beds" in the hospital, of which there appear to be seventy-seven. There is also an article on "How to Become a Ventriloquist," by Sergt. G. Gillson, S.A.I., in which we are reminded that Echo is aitchless, some photographs, an account of the recent Royal visit to the hospital, a study in "Army Hieroglyphics," topical notes, the inevitable "What we Want to Know," and the "Chaplain's Corner." So there is a varied content. What an amusing article might be written, by the way, on "How to Avoid Profanity"! There is also an article on the workshops.

### THE SCARCITY OF SUGAR SUBSTITUTES.

Some weeks ago it was suggested in The Hospital that hospital buyers might with advantage secure a stock of honey, since the price of everything that could possibly be used as a sweetening agent was likely to rise to extravagant prices. Those who acted on this advice did wisely, for the value of honey has since risen substantially, and there are prospects of pronounced scarcity and famine prices. At the recent public sales in Mincing Lane the offerings of honey were eagerly snapped up at figures something like three times as high as those ruling before the war; in fact, the value of foreign honey is now something like double the price of sugar. Saccharin is worth twenty guineas per lb., about 800 times the price of sugar and only some 500 times as sweet! The other chief sweetening agent that can be used in place of sugar is glucose; the price of this is tending upwards, and hospital buyers who look ahead will not omit to consider the expediency of buying a supply of glucose before it has time to become much dearer. It goes without saying that buyers will make sure of obtaining a glucose of good quality and take care that it is as free as possible from impurities.

### THIS WEEK'S DRUG MARKET.

The tendency of values is still in an upward direction, but in spite of this a fair business continues to be done. Extremely high prices have been paid for calumba root. Honey changes hands at extraordinary figures. Kola nuts are dearer. Business in quinine is restricted, but prices have a strong upward tendency. The new and higher prices for resublimed iodine, potassium iodide, sodium iodide, and iodoform came as a surprise to the drug market. Almond oil is again dearer. Casein, which is largely used in the manufacture of so-called "nerve foods," is scarce and dearer. The upward movement in the price of sodium bicarbonate continues. In sodium salicylate and salicylic acid there is practically no change, and there seems to be no difficulty in obtaining adequate supplies. Potassium permanganate is as difficult as ever to obtain, and prices are rather higher. Methyl salicylate (artificial oil of wintergreen) is offered at rather lower rates. Phenacetin and saccharin also have a slightly downward tendency in price.

### TO OUR READERS.

Contributions are specially invited from any of the readers of The Hospital. They should deal with topical subjects and news or incidents illustrating any department of hospital work, its progress or difficulties. They must be authenticated for the information of the Editor only. The minimum payment if published is 5s. There is no hard-and-fast rule as to space, but paragraphs of about twenty lines in length are preferred.

